# Experimental quantification of pollen with DNA metabarcoding using ITS1 and trnL

**DOI:** 10.1038/s41598-020-61198-6

**Published:** 2020-03-06

**Authors:** Sandra Baksay, André Pornon, Monique Burrus, Jérôme Mariette, Christophe Andalo, Nathalie Escaravage

**Affiliations:** 1 Laboratoire Evolution and Diversité Biologique EDB, CNRS, UMR 5174, Université Toulouse III Paul Sabatier, F-31062 Toulouse, France; 2 Plate-forme Bio-informatique Genotoul, Mathématiques et Informatique Appliqués INRA, UR875, Toulouse, F-31320 Castanet-Tolosan France

**Keywords:** Plant biotechnology, Plant ecology, Plant genetics

## Abstract

Although the use of metabarcoding to identify taxa in DNA mixtures is widely approved, its reliability in quantifying taxon abundance is still the subject of debate. In this study we investigated the relationships between the amount of pollen grains in mock solutions and the abundance of high-throughput sequence reads and how the relationship was affected by the pollen counting methodology, the number of PCR cycles, the type of markers and plant species whose pollen grains have different characteristics. We found a significant positive relationship between the number of DNA sequences and the number of pollen grains in the mock solutions. However, better relationships were obtained with light microscopy as a pollen grain counting method compared with flow cytometry, with the chloroplastic *trnL* marker compared with ribosomal ITS1 and with 30 when compared with 25 or 35 PCR cycles. We provide a list of recommendations to improve pollen quantification.

## Introduction

Environmental DNA metabarcoding is a molecular method that consists of investigating environmental DNA samples made of complex mixtures of genomes from numerous organisms^1^. Due to new sequencing technologies and bioinformatics tools, metabarcoding has been increasingly used to identify taxa in environmental samples^1^ to monitor biodiversity^2–4^, to investigate ecosystem functioning^5^ and interaction networks^6–8^, in both aquatic and terrestrial ecosystems. Nevertheless, its reliability in quantitative approaches, which depend on the match between counts of high-throughput sequence reads and the amount of sampled biological material^2^, is still the subject of debate^9,10^. While taxon identification can reveal individual diet breadth^11^, species richness, and the composition of habitats^2^, communities^12^ and ecological networks^4^, taxon quantification provides knowledge on species evenness in those habitats, communities and diets or on the level of individual or species specialization in networks, all of which is very useful in ecological studies. Research on pollination and knowledge of the quantities of pollen transported by pollinators allow for the estimation of plant-pollinator interaction strength and hence it gives a more realistic representations of networks than those made possible using traditional approaches such as observing visits to plants by pollinators^9,13^.

Metabarcoding has been used in pollen studies to identify pollen in honey^14–16^, insect loads^6–8,17^, insect nests^18^, airborne samples^19^, and to quantify pollen abundance across various sample types. Several studies found significant positive relationships between pollen abundance (estimated using light microscopy) or pollen DNA quantities, and the abundance or the frequencies of high-throughput sequencing reads in experimental samples^10,16,17,20,21^, airborne samples^22,23^, insect pollen loads^21,24–27^ or in brood cells of solitary bees^28^. Conversely, other studies found low or no significant pollen-sequence abundance relationships when using ITS2 markers applied to pollen provision in bee corbicula containing huge amounts of pollen^17,21^.

Many factors associated with handling samples, technical processes, or the biological material itself can affect the accuracy of metabarcoding quantification. Some factors, related to pollen (size, structure), species DNA (gene copy number) and the characteristics of DNA markers (nuclear *vs* plastid), cannot be corrected whereas it may be possible to reduce the impact of others. Establishing relationships between pollen quantity and DNA sequence abundance requires accurate estimates of the concentration of pollen in the samples used for DNA extraction. However, pollen concentration measurements made using light microscopy, and pollen DNA isolation are usually performed on different subsamples which possibly result in differences in pollen concentrations as a result of pipetting artefacts. In addition to the possible artefacts that occur during PCR and preparing sequencing, these may cause significant variation among replicates and artificially affect the relationship between the concentration of pollen and sequence abundance. An alternative approach which, to our knowledge, has not yet been tested, is counting pollen grains in the DNA isolation pellets. Since at this stage of experimental processing there is no longer a risk of DNA contamination in the laboratory, pollen counting could be automated and applied to the whole pollen population, rather than to a small fraction, as is the case in traditional methods using microscopy. However, post-PCR counting would preclude crushing pollen grains for DNA extraction, which, in any case, is not required for efficient DNA isolation^20^. Efforts are also needed to reduce the accumulation of spurious sequences, chimeras and Taq polymerase inhibitors, during PCR and sequencing processes^29^, which are expected to fluctuate with the genetic markers, the PCR and sequencing conditions^21,30^ and the reagents used. Many studies which investigated artificial^26,30,31^ or natural pollen mixtures^17^ used samples with huge pollen concentrations (from 30,000 to potentially more than 1,000,000 pollen grains) stored in a single bee corbicula^30,31^ or several grams of pollen^26,27^. Such large quantities of pollen could release large amounts of DNA polymerase inhibitors^32^ and cause PCR dysfunction. It would thus be useful to design experiments with lower pollen amounts, for instance, in the range of those involved in pollination, typically from a few to several thousand grains^33,34^. All these potential biases can be exponentially amplified during PCR, increasing the variability of the data set^31^, reducing the strength of statistical tests and affecting the relationship between pollen concentrations and sequence abundances.

We investigated relationships between the number of pollen grains and sequence counts abundance obtained by high-throughput sequencing, using two species (*Chrysanthemum* sp. - *Asteraceae*, and *Hippeastrum* sp. - *Amaryllidaceae*) of which the pollen has very different characteristics (size, exin structure, DNA content)^35–39^. We analysed two plant specific loci, the internal transcribed spacer region 1 (ITS1, ≈ 300 bp) of the nuclear ribosomal region, and the plastidial P6-loop of *trnL* (UAA) intron (≈75 bp), efficient for investigating potential degraded DNA, respectively, hereafter referred to as ITS1 and *trnL*. The loci were amplified using three different PCR conditions (25, 30 and 35 cycles). The number of pollen grains in each subsample was estimated before DNA isolation using light microscopy. Moreover, as we demonstrated in previous work^20^ that the mechanical pollen disruption did not give higher DNA yield, we used flow cytometry to count uncrushed pollen grains in the extraction pellets after enzymatic DNA isolation. Our main objectives were to investigate, for pollen quantities in the range of insect pollen loads involved in plant pollination: (1) whether using flow cytometry on extracted solutions provides more accurate estimates of pollen grain abundance than light microscopy, and consequently, a stronger relationship between the number of pollen grains and sequence abundance; (2) how the relationship is affected by the number of PCR cycles, the type of markers and plant species with different pollen characteristics.

## Results

### Read scores of trnL and ITS1 amplifications

We obtained 2,699,831 *trnL* reads (360 samples), of which 56.68% were assigned to *Hippeastrum* sp. (HIP) and 43.32% to *Chrysanthemum* sp. (CHR). The total number of ITS1 reads (360 samples) reached 302,934, most of which (78.59%) were assigned to CHR.

The 25 PCR cycle amplification conditions produced relatively few *trnL* (on average, 98 and 19 reads for HIP and CHR, respectively) and ITS1 reads (on average, 14 and 169 sequences for HIP and CHR, respectively). Compared to 25 PCR cycles, the numbers of *trnL* sequences at 30 and at 35 PCR cycles were 23 and 235 multiplied in HIP and 65 and 954 multiplied in CHR, respectively. Compared to 25 PCR cycles, the number of ITS1 sequences at 30 and 35 PCR cycles respectively, was multiplied by 24 and 56 in HIP and by 11 and 12 in CHR, respectively. Therefore, ITS1 amplification was lower than that of *trnL* and increased slightly with an increase in the number of PCR cycles.

### Microscopy and cytometry pollen counts

**Figure 1:**
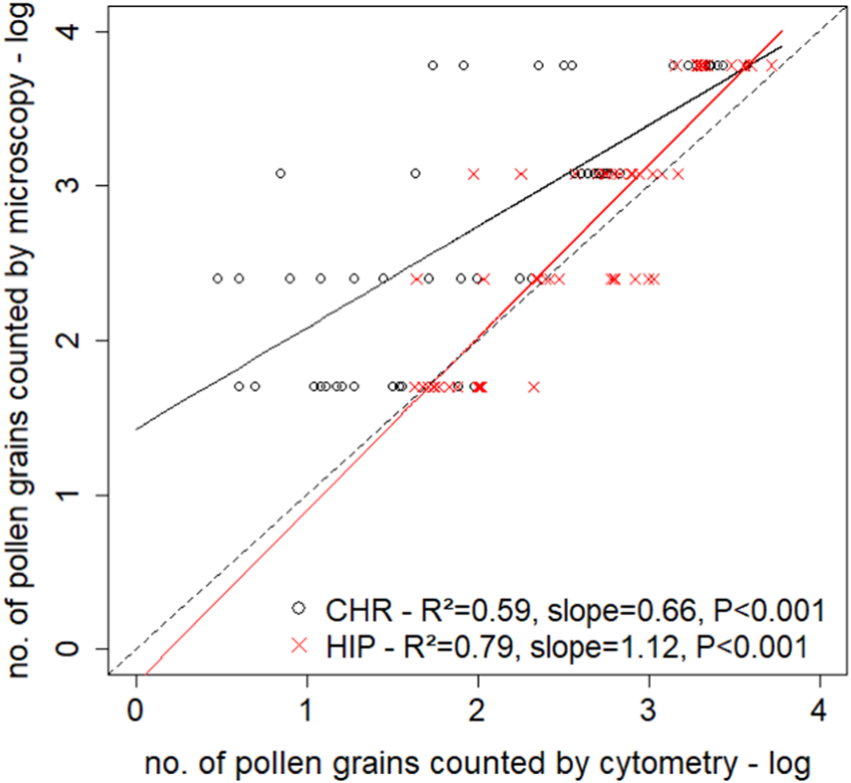
Relationships between the number of pollen grains in *Hippeastrum* sp. (red lines) and in *Chrysanthemum* sp. (black lines) estimated by microscopy and by flow cytometry.

We found a highly significant positive relationship between the numbers of pollen grains estimated by microscopy and flow cytometry (Fig. 1). However, the relationship was clearly better for HIP (higher R^2^; line slope close to 1) than for CHR. Flow cytometry tended to detect fewer CHR pollen grains than microscopy, especially in samples with low pollen abundance (Fig. 1).

### Relationships between DNA sequence abundance and pollen grain abundance estimated using light microscopy

**Figure 2:**
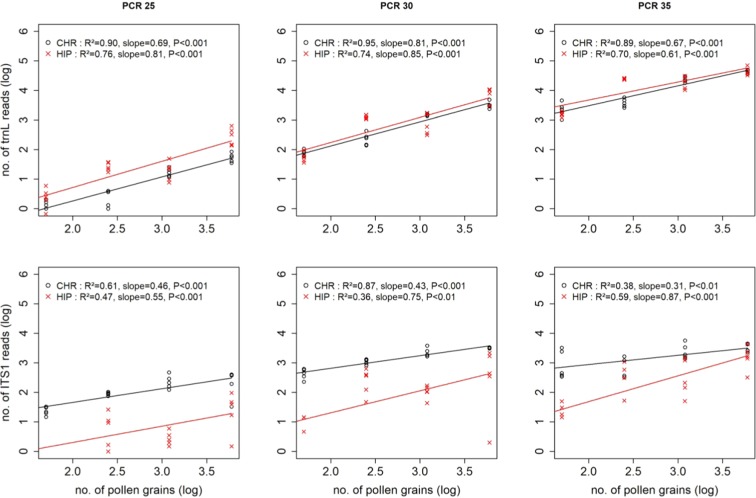
Relationship between the number of *trnL* and ITS1 reads and the number of pollen grains (log transformed) in *Hippeastrum* sp. (red lines) and in *Chrysanthemum sp*. (black lines) estimated by light microscopy in three different PCR cycles.

For both markers and regardless of the number of PCR cycles, ANCOVA linear model (lm) revealed highly significant positive relationships between the number of sequences and the number of pollen grains (Table 1; Fig. 2). However, the relationships (Fig. 2) were generally stronger (R^2^ always ≥ 0.70) and less variable for *trnL* (CHR: 0.89 ≤ R^2^ ≤ 0.94; HIP: 0.70 ≤ R^2^ ≤ 0.76) than for ITS1 (R^2^ generally ≤ 0.61; CHR: 0.38 ≤ R^2^ ≤ 0.87; HIP: 0.36 ≤ R^2^ ≤ 0.59). For *trnL*, the slope of the relationship ranged from 0.61 to 0.85, that is a 1 log increase in the number of pollen grains, a 0.61-to-0.85 log increase in sequence quantity (Fig. 1). For ITS1, the values were generally both lower and more variable (0.31 ≤ slope ≤ 0.87) across PCR conditions and species. The two plant species had very similar (*trnL*) or quite similar slopes (ITS1), except HIP at PCR35 for ITS1, which had a steeper slope than CHR.

**Table 1:** ANCOVA model of the effects of plant species (HIP or CHR) and pollen quantity (log transformed) counted using light microscopy or flow cytometry on the quantity of ITS1 and *trnL* reads obtained with 25, 30 or 35 PCR amplification conditions (PCR25; PCR30, PCR35 respectively).

Source of variation	PCR25			PCR30			PCR35		
	Sum sq	Df	F value	Sum sq	Df	F value	Sum sq	Df	F value
**Counting using microcopy**									
**ITS1**									
Plant species	15.51	1	102.30***	16.19	1	48.00***	7.10	1	31.20***
Pollen quantity (log)	5.82	1	38.39***	8.22	1	24.37***	8.41	1	36.99****
Plant species x Pollen quantity	0.04	1	0.29 ns	0.60	1	1.79 ns	1.88	1	8.25**
Residuals	5.30	35		0.34	36		8.19	36	
***trnL***									
Plant species	2.60	1	24.12***	0.20	1	2.07 ns	0.21	1	2.96 ns
Pollen quantity (log)	17.31	1	160.95***	16.60	1	168.57***	9.84	1	138.26***
Plant species x Pollen quantity	0.03	1	0.29 ns	0.01	1	0.06 ns	0.03	1	0.36 ns
Residuals	3.87	36		3.55	36		2.56	36	
**Counting using flow cytometry**									
**ITS1**									
Plant species	15.51	1	90.47***	16.19	1	39.10***	7.10	1	25.61***
Pollen quantity (log)	4.79	1	27.94***	5.26	1	12.71**	6.38	1	23.00***
Plant species x Pollen quantity	0.38	1	2.19 ns	0.80	1	1.94 ns	2.12	1	7.67**
Residuals	6.00	35		14.91	36		9.98	36	
***trnL***									
Plant species	2.29	1	8.83**	0.18	1	0.67 ns	0.23	1	1.47 ns
Pollen quantity (log)	8.76	1	33.81***	8.27	1	31.28***	5.33	1	33.78***
Plant species x Pollen quantity	0.86	1	3.33 ns	0.95	1	3.59 ns	0.42	1	2.68 ns
Residuals	7.77	30		7.93	30		4.73	30	

ns: not significant; **P <0.01; ***P <0.001.

There was also a significant species effect in each PCR cycle for ITS1 but only at PCR25 for *trnL* (Table 1). Namely, CHR had slightly fewer *trnL* sequences at PCR25 but many more ITS1 sequences than HIP in all PCR cycles (Fig. 2). The two plant species had very similar (*trnL*) or quite similar pollen-sequence relationship slopes (ITS1), except HIP at PCR35 for ITS1 which had a steeper slope than CHR. Overall, there was no pollen quantity–plant species interaction, except for ITS1 at PCR35.

Finally, while the number of *trnL* sequences tended to steadily increase with increasing PCR cycles (Fig. 2), the number of ITS1 hardly increased between PCR30 and PCR35. Thus, the amplification efficiency of ITS1 decreased with the number of PCR cycles, however, without fundamentally altering the relationships between pollen and sequence abundances.

### Relationships between DNA sequence abundance and pollen grain abundance estimated using flow cytometry

**Figure 3:**
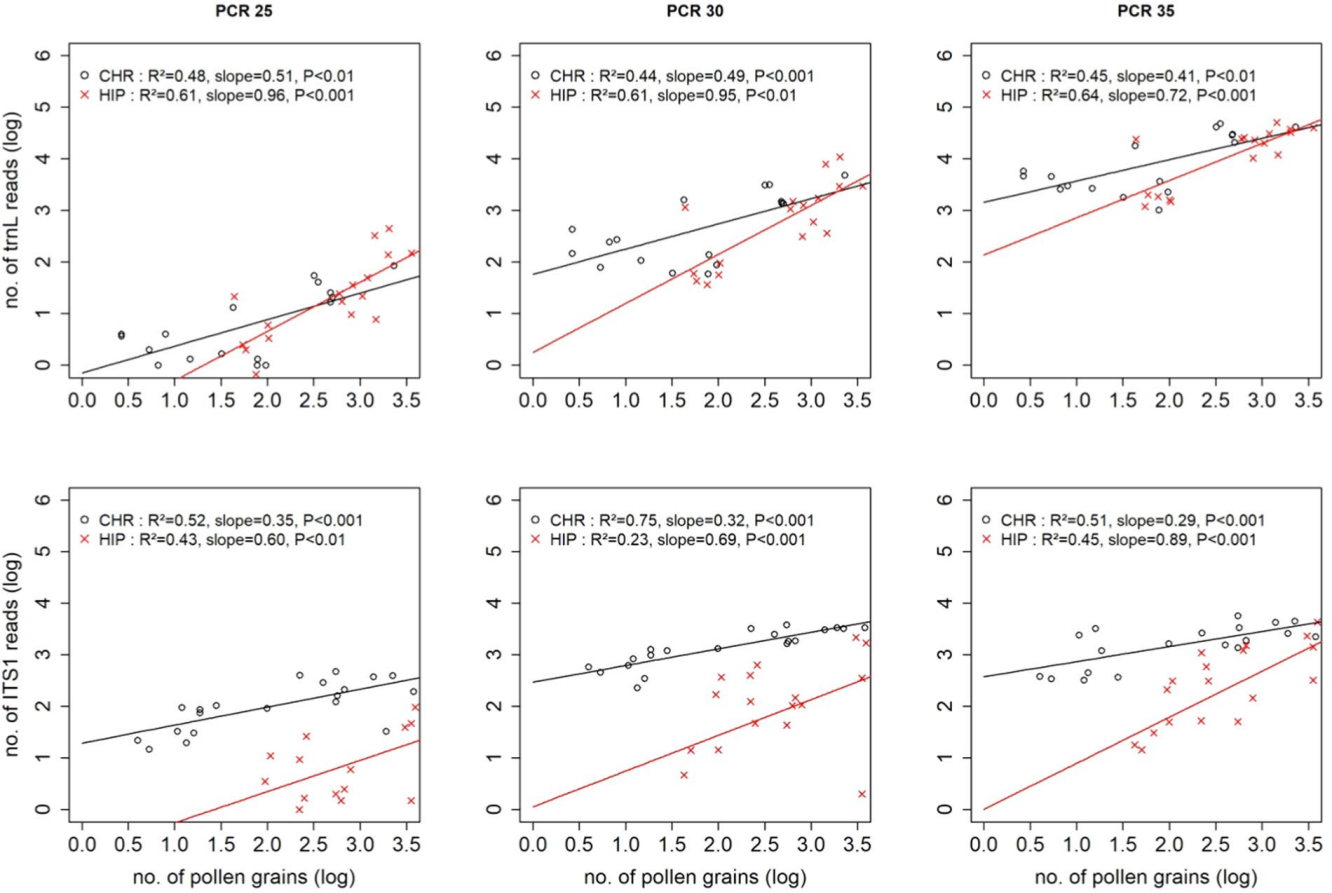
Relationship between the number of *trnL* and ITS1 reads and the number of pollen grains (log transformed) in *Hippeastrum* sp. (red lines) and in *Chrysanthemum* sp. (black lines) estimated by flow cytometry in three different PCR cycles.

The ANCOVA linear model highlighted very similar trends in the relationship as microcopy estimates (Table 1; Fig. 3) i.e.: (i) a strong significant effect of the number of pollen grains on the number of ITS1 and *trnL* sequences; (ii) a species effect, mostly for ITS1; (iii) generally no pollen grain x plant species interactions, and (iv) less variability in R^2^ and slope for *trnL* than for ITS1. On the other hand, flow cytometry pollen estimates gave weaker predictions (lower R^2^ and line slopes) of *trnL* sequence quantities, thus showing that flow cytometry was generally less efficient than microscopy in counting pollen grains.

## Discussion

In this study, we investigated whether it is possible to predict the abundance of DNA sequencing reads from the quantities of pollen grains and if so, how accurately, and how the prediction is affected by the methodology used to estimate pollen abundances in mock solutions, PCR conditions, plant species and the type of markers (nuclear versus plastid). In agreement with previous studies^20–22,25,40^ but in contrast to others^17,26,30,31^, we found a significant, and often strong positive relationship between the number of DNA sequences and the number of pollen grains in the mock solutions. However, the strength of the relationship was influenced by the pollen counting methodology, the marker, the species and the number of PCR cycles.

### Efficiency of microscopy vs flow cytometry in counting pollen grains

Usually, in experiments which aim to evaluate the potential of metabarcoding for pollen quantification, the estimates of pollen abundance in suspension and the DNA extractions have been performed using different subsamples^17,20–22,25–27,30,31,40^, resulting in unavoidable variation in pollen concentrations. Moreover, since counting pollen grains under a light microscope is very time consuming and as mock solutions often have high concentrations of pollen, only small subsamples and a small proportion of microscope slides (and consequently of pollen population) are usually inspected^23^. Such methodological problems may then be further amplified by PCR, thus blurring the relationship between sequence and pollen abundance. In an attempt to reduce these biases, in every pollen sample based on microscopy counting, we counted pollen after DNA isolation using flow cytometry. While DNA isolation and flow cytometry were performed on the same mock solution and since more than 80% of the stock solution was counted, we expected flow cytometry to provide the best estimates of pollen grain quantity, and therefore, the best sequence-pollen grain abundance relationships. Unexpectedly, stronger relationships were found using light microscopy, showing that it provided better estimates of pollen abundance than flow cytometry. Moreover, the efficiency of flow cytometry appeared highly species-dependant. Indeed, both microscopy and flow cytometry provided similar estimates of HIP pollen grain abundance, but flow cytometry largely underestimated the abundance of the smaller CHR pollen grains, especially in the less concentrated mock solutions (50–250 grains). We observed that, despite the fact that pollen grains were not crushed before DNA extraction many CHR grains were in fact fragmented after DNA extraction. Fragments were then likely confused in spectrograms with the uncounted myriad of tiny biological and mineral particles present in the mock solutions. On the other hand, even though partially destroyed, the bigger pollen grains of HIP could still have been detected and counted. Moreover, due to the absence of lacunae and only one aperture, HIP pollen grains were possibly less sensitive to fragmentation than CHR pollen grains.

### Effects of molecular markers and PCR conditions

We found that for *trnL*, very accurate prediction of DNA read abundance was obtained from pollen grain quantities estimated by light microscopy, with determination coefficients and slopes generally higher than those found in the literature with the same markers^40^ or with other markers^17,21,26,30,31^. Moreover, except at PCR25, the good relationship was conserved across species and PCR cycles and *trnL* sequence abundance increased steadily with increasing PCR cycles. These positive results confirmed that: (i) light microscopy counting of pollen grains after DNA extraction was a suitable method to prepare mock solutions and replicates, (ii) *trnL* amplification was not subject to important PCR biases, and (iii) the degree of repeatability of high-throughput sequencing was high^41^.

The relationship between pollen–DNA sequence counts and ITS1 was also highly and positively significant, in the same range as that obtained by other authors^20,27^ but with less accuracy (lower determination coefficients and slopes) and higher variability across species and PCR conditions than with *trnL*. Although HIP has higher pollen DNA content^42^ than CHR^36^, it produced fewer ITS1 sequences than CHR, whereas both species produced similar quantities of *trnL* sequences regardless of the quantity of pollen and PCR conditions. Furthermore, in contrast to what we observed for *trnL*, ITS1 abundance did not increase steadily with the number of PCR cycles and remained almost stable between PCR30 and PCR35, although with a modification in the relationship between the pollen and sequence abundances. These results suggest that: (i) the amplification efficiency and copies of *trnL* in pollen from the two species is comparable despite the fact that the number of plastids may vary across species^43^, (ii) HIP had fewer ITS1 copies in its nuclear genome, lower amplification or sequencing efficiency than CHR; (iii) the processes involved in these alternative causes would have been primarily influenced by DNA characteristics rather than by DNA quantities *per se*.

The observed differences between species, markers and PCR conditions could be related to the GC content in *trnL* and ITS1 sequences. Indeed, high GC contents can inhibit amplification^44^ due to primer mis-annealing, *Taq* DNA polymerase errors, gene mis-amplification and the synthesis of chimeras^45^. High GC-richness can also alter sequence reading during sequencing^46^. Analysing rDNA genes, Pinto & Raskin (2012) found that sequence counts were inversely proportional to the GC content of the samples (R = 0.78). We consistently observed that CHR had 60% GC in ITS1 and only 30% in *trnL* and that HIP had 10% more GC in ITS1 but only 1% more GC in *trnL* than CHR. Furthermore, amplification and sequencing using Illumina technologies are more effective for short DNA fragments and cause less variability in amplicon length than for longer fragments^47^. Together, the difference in GC-richness between markers and species and in sequence length probably explained the lower yield of ITS1 sequences compared to yields of *trnL* sequences in general and for HIP compared to CHR in particular, while both species had almost equal quantities of *trnL* sequences. Moreover, the accumulation of GC-induced inhibitors with PCR cycles^32,45^ is likely responsible for the fact that the number of ITS1 sequences did not increase beyond PCR30, a trend that was not observed in *trnL*.

### Recommendations and methodological considerations when using metabarcoding for pollen quantification

Despite its high potential, we know only three other studies that (successfully) used *trnL* in pollen quantification^20,22,40^. Therefore, our findings call for further studies to determine whether *trnL* can be applied to many other plant species and routinely used to take greater account of interaction strength in pollination network studies. Kraaijeveld *et al*., (2015) noted that *trnL* also had higher discriminatory power than other standard markers such as ITS, *rbcL* and *matK*. It is worth noting that, probably due to their relatively low GC content, plastid loci such as *trnL*^20,22,40^ (≈35% GC content), *rbcL*^26,40^ (≈42% GC content), *matK* (≈35% GC content) or *trnT*^23,48^ (≈25% GC content) may provide a better estimation of pollen number than nuclear markers (ITS1 and ITS2 ≈ 60% GC content). However, the drawbacks of ITS could to some extent be alleviated^49^ by using high-fidelity DNA polymerases such Phusion High-Fidelity DNA polymerase^25,40^ or Herculase II Fusion DNA polymerase (the present study), 3% (our study) or 5% DMSO^50^ and by applying low primer annealing temperatures^45^. The detrimental impact of the accumulation of polymerase inhibitors could be reduced by increasing the number of PCR steps (3 to 5 successive PCRs) and by diluting a subsample of the previously obtained amplicons in fresh reagent mixtures at each step^45^. However, this method would be costly and time-consuming and not practical for high input studies.

Some experimental studies^30,31^ or studies whose goal was to understand patterns of floral resource use in bees analysed huge quantities of pollen^21,40^ sometimes corresponding to several pollen pellets. The resulting high DNA amounts could have led polymerase inhibitors to accumulate in solutions with increasingly negative consequences for PCR amplification and thus explain the low correlation or the lack of a correlation between sequence counts and pollen quantities reported in some studies^21,26,30,31^.

With 30 PCR cycles, both *trnL* and ITS1 markers provided the highest relationship slope and the best sequence-to-pollen prediction (*trnL*) while the effects of plant species either alone (PCR25/*trnL*) or in interaction with pollen amounts (PCR35/ITS1) were minimised. Moreover, for the low-pollen amount samples, PCR25 had the disadvantage of producing few sequences, which could be confused with contamination and be removed from the data set by protocols designed to remove eDNA contamination (airborne pollen or non-pollen plant tissues deposited on insect bodies^8^). A dual-indexing strategy (dual-tagging PCR amplification and single-run sequencing) has sometimes been used to identify pollen by metabarcoding^21,28,30,40^, but this approach is not recommended for pollen quantification^28^, especially if the single–run sequencing involves multi-locus analysis. In practice, better quantification results have often been obtained with single-indexing PCR amplification and single-locus sequencing^20,22,23,26,27,48^.

To sum up, to improve pollen quantification, we recommend that: (1) unless special care has been taken to prevent pollen break out, to avoid using flow cytometry, or trying to perform flow cytometric counting before DNA extraction; (2) in the case of very high pollen abundances, as typical in bee corbicula, to either dilute samples to obtain solutions with a few thousand pollen grains or to use an alternative method such as PCR-free genome-skimming^51^; (3) to use a multi-locus approach including short plastid markers with low GC content, and (4) to use high-fidelity Taq DNA polymerases (such as Phusion High-Fidelity or Herculase II Fusion DNA polymerases), 3% or 5% DMSO, and a low primer annealing temperature to reduce some of ITS marker weaknesses; (5) to apply, at least for *trnL* and ITS1, a 30 cycle PCR; (6) to use single-locus sequence analysis rather than multi-locus sequencing; and finally, (7) to normalize the amplicon concentration before sequencing in order to reduce sequencing artefacts due to very different DNA amplicon concentrations^27,52^.

While we only investigated two plant species, additional studies are required to determine whether our findings can be generalised to many other species. Moreover, we did not consider species mixtures. Some studies did not detect any pollen-mixture effect^22^ but others did. For instance, Bell *et al*. (2018) and Richardson *et al*. (2015) found under- or over-representation of some species in sequencing products compared to pollen populations estimated using light microscopy. In studies using plant mixtures, an effect of species identity on the sequence-pollen DNA abundance correlation has been observed, but without deleting the sequence-pollen counts correlation^20^.

Furthermore, using ITS1 and *trnL*, Pornon *et al*.^20^ found highly significant positive correlations between the number of insect visits to plant species (more than 23 species in mixtures) and the number of their sequences in sequencing products. This occurred even though visits to flowers do not usually accurately reflect pollen transport by insects^53^. So, we believe our findings are not an exception but that the question of pollen quantification in mixtures deserves further investigations that take all the recommendations we suggest here into account.

## Material and methods

### Plant models

We investigated variation in the number of reads produced by 50, 250, 1,200, 6,000 pollen grains belonging to the ornamental cultivars, *Hippeastrum* sp. (HIP, Amaryllidaceae) and *Chrysanthemum* sp. (CHR, Asteraceae). We were unable to identify the species to which the plants belonged, likely because they were the products of many species and cultivar crossings (*H. striatum*, *H. mandoni*, *H. papilio* and *C. dichroum*, *C. indicum*, *C. chanetii*, *C. x morifolium*). The plants were chosen because they are widely sold in gardening stores, they produce large amounts of pollen (for more details, see a previous study^20^) in winter when native species are not in flower and because their pollen has strikingly different characteristics. CHR has small (20 µm to 35 µm diameter), echinate pollen grains with a tectate ectoexine with large lacunae and three apertures^38^, and a 2 C value of 11.87 pg DNA^36^. HIP pollen grains are bigger (50 µm to 100 µm in diameter), with a semitectate ectoexine and one aperture^35^. Their DNA content ranges between 2 C = 13.35 pg and 2 C = 17.09 pg^42^. These differences between and within species in pollen grain structure and DNA content may potentially influence the amount of DNA extracted and the number of sequencing reads produced by each pollen grain.

### Pre- and post-extraction pollen counts

Pollen stock solutions were obtained for each species by vigorously shaking fresh stamens in 10 mL sterilized tubes, kept sealed at 4 °C, then adding 3 mL of lysis buffer CF solution (Nucleospin Food Kit, Macherey-Nagel) to each tube and mixing thoroughly. The concentration of pollen grains in each stock suspension was estimated (pre-extraction estimations) by counting pollen grains in 10 μl (HIP) or 2 μl subsamples (CHR, due to the higher pollen abundance) under a light microscope, with seven replicates per stock suspension. Based on the known pollen concentration of the stock suspensions, we took subsamples of each species each containing 50, 250, 1,200, 6,000 pollen grains with five replicates (giving a total of 40 mock solutions for the two species). After DNA extraction and isolation, we performed an additional pollen grain count (post-extraction estimations) of each mock solution using flow cytometry (CyFlowSpace Sysmex). As the samples with ITS1 and *trnL* are not the same, two batches of 40 pellets were counted for the two markers, containing respectively the two types and the four quantities of pollen. To prevent rapid pollen sedimentation, extraction pellets with empty pollen grains were re-suspended in 1 ml of 60% glycerol before counting using flow cytometry.

### DNA extraction, PCR amplification and sequencing

Total DNA extraction of mock solutions was performed with the DNeasy Plant Mini kit (Qiagen) according to the protocol of Pornon *et al*.^20^. *trnL* and ITS1 markers were then amplified at 25, 30, 35 PCR cycles with three replicates per PCR cycle condition (giving a total of 720 PCR products, corresponding to five extraction samples for each quantity of pollen x 4 pollen quantities x 3 PCR conditions x 3 PCR replicates x 2 plant species x 2 markers). For both *trnL* and ITS1 amplification, PCR reactions were performed in a 25 µl reaction volume containing 5 µl 5x Herculase II reaction Buffer, 25 mM each dNTP, 0.4 µM of each tagged PCR primer, 0.25 µl Herculase II fusion DNA polymerase, 2 µl DNA and distilled water. After reviewing the PCR protocol from Pornon *et al*.^20^ and optimizing the annealing temperatures at 55 °C and 50 °C for *trnL* and ITS1 respectively, the PCR programs were: 2 min denaturation at 95 °C; followed by 25, 30 or 35 cycles (20 s denaturation at 95 °C, 20 s annealing at 55 °C (50 °C for ITS1), 30 s elongation at 72 °C) and a final elongation at 72°C for 3 min. For ITS1, 3% DMSO was added in the reaction solution to increase Taq polymerase specificity^50^. PCRs were performed in the Thermal Cycler GeneAmp PCR System 9700 (Applied Biosystems) and each PCR product was visualized on 1% agarose in TAE 0.5X buffer and quantified on the QuantStudio 6 Flex Real-Time PCR System (Applied Biosystems). Two libraries (one for each marker) were generated using 2 µl of each PCR product following the manufacturer’s guidelines for the Illumina TruSeq Nano kit, except that no sonication was performed. Libraries were sequenced using the MiSeq Illumina technology, 2 × 250 paired-end run for ITS1 and 2 × 75 paired-end run for P6-loop of *trnL*, using the NGS core facility at the Génopole Toulouse Midi-Pyrénées (www.get.genotoul.fr).

### Sequence analysis and identification of plant taxa

We built our own barcoding reference library using barcodes from Pornon *et al*.^20^ and completed with barcodes of all species belonging to the *Chysanthemum* and *Hippeastrum* genera obtained from the EMBL database, using the ecoPCR function of the OBITOOL package^54^ and following the OBITOOL pipelines (git.metabarcoding.org/obitools/ecopcr/). Sequence treatment followed a step-by-step analysis procedure of the OBITOOL protocol. Paired-end reads were assembled using the Illumina paired-end utility that aligns the two reads and returns the reconstructed sequences. Sequences of low alignment quality (<40%) were discarded. Each sequence was affiliated to its corresponding sample using the ngsfilter command and dereplicated into unique sequences using the obiuniq command. As some of these sequences may contain PCR and/or sequencing errors, as well as chimeras, they have to be discarded using the obigrep command while keeping sequences more than 20 bp long and with a count equal to or greater than 10 sequences. As a final denoising step, we kept sequences with no variants, with a count greater than 5% of their own count (command obiclean). Then, a single taxon was assigned to each sequence using the ecoTag program that compared the sequences produced to our taxonomic reference library. When assigning our two target plant species, a best match score >95% was allowed for each marker.

### Statistical analysis

To compare the two methods of estimating the number of pollen grains in HIP and CHR, we fitted a linear regression model to the number of pollen grains estimated by flow cytometry using the number of pollen grains estimated by cytometry as predictive variable. To analyse the relationship between the number of pollen grains (estimated by microscopy and by flow cytometry, respectively) and the number of sequence counts, we fitted an ANCOVA linear model with the lm function of the R base package^55^. Independent statistical analyses were performed for each combination of markers (ITS1 or *trnL*) and PCR conditions (25, 30 or 35 cycles). We tested the fixed effects of the logarithm of the pollen quantity, plant species (HIP and CHR) and their interaction on the mean number of sequence reads averaged over the three PCR replicates. Different analyses were performed for either pre-extraction or post-extraction estimates of pollen grain quantities. When the interaction between plant species and pollen amount was not significant with respect to sequence abundance, it was removed from the model and simple effects of either plant species or pollen quantity were tested (type II sum of squares). Normality of the residual distribution was systematically checked and, if necessary, a log transformation was applied to the response variable.

## Data Availability

The data that support the findings of this study will be available in a public repository.
